# Historical remarks on Martin Kirschner and the development of the Kirschner (K)-wire

**DOI:** 10.4103/0970-0358.41122

**Published:** 2008

**Authors:** W. Huber

**Affiliations:** Consultant Trauma, Hand and Micro surgeon, UKH Linz Trauma Center, Linz, Austria

Over decades the K-wire (or Kirschner-wire) has been an extremely versatile tool in the hands of plastic and orthopaedic surgeons who use it for temporary or definitive osteosynthesis, temporary joint transfixation or for guidance for other implants like cannulated screws. The wire is named after a famous German surgeon, Martin Kirschner. He was born on October 28th 1879 in Breslau, (today Wroclaw, Poland). With the exception of his father - a solicitor - all of his ancestors from the 18^th^ century onwards had treated wounds or become surgeons. When he was 14 years old, Kirschner moved to Berlin. He gained admission to the medical school in Freiburg at his second attempt. His initial failure caused him some disappointment. He later continued his education in Strasbourg (today in France). He finished his pre-clinical studies there with A-grades in all subjects, a unique achievement at the time. He continued his medical training in Strasbourg, and Munich before graduating in 1904.

M. Kirschner began his career in general medicine in Berlin but he was soon attracted to surgery and started working with Payr in Greifswald in 1908. Payr was a famous surgeon, with whom he moved on to Koenigsberg (formerly in Prussia, now capital of the Kaliningrad province of Russia) in October 1910. Three years later he started work in Leipzig (Germany). He first experienced war surgery during a Red Cross expedition to Sofia and Adrianopel in 1912/13. Later he worked as a surgeon on the Western Front in the First World War during 1914-15. He then continued to work in Koenigsberg and was appointed as head of the surgical department and professor in 1916, the same year that he was married. In Koenigsberg Martin Kirschner performed the first successful pulmonary embolectomy in 1924. From 1927 to 1934 he was head of the department of surgery in Tübingen (Germany) and in 1934 he was elected President of the German Society of Surgery. In that year he moved to Heidelberg (Germany). In 1942 he had an operation for a gastric ulcer, which was found to be malignant with liver metastases and local infiltration of the pancreas. He died on August 30^th^ 1942 aged 63 years.[1] In 1943 Payr published an obituary in which he described M. Kirschner as a Tatenmensch (“man of action”) and described his attitude towards colleagues as respectful but rarely kind. Kirschner was said to be a sharp critic, who deeply despised lack of logic or objectiveness.[2]

His scientific research and academic interests addressed topics which in our days are covered by several specialities such as general surgery, orthopaedic surgery, neurosurgery, urology, anaesthesiology and even plastic surgery. But it is not only his contribution to orthopaedic surgery that still influences our daily routine as surgeons. His description of tourniquet application and the invention of individually adjustable spinal anaesthesia are ever present in our daily routine, even if not linked to Kirschner by eponym.[3]

Kirschner published 249 articles in medical journals, contributed to 8 textbooks on almost every aspects of surgery and edited five medical journals. One of his first academic interests was of tissue defects and in proving the versatility of autologous free fascia transfer. His skill in general and vascular surgery contributed significantly to cancer surgery of the stomach, colon and rectum. He showed that the stomach could be mobilized without vascular compromise and could therefore be used for oesopaghoplasty. He also modified the Bassini technique for inguinal hernia repair in order to reduce the recurrence rate. In 1924 he caused a sensation when he performed the first successful pulmonary artery embolectomy He modified the technique of craniotomy that was used at the time and contributed to neurosurgery with his proposals for the treatment of cortical epilepsy. But his impact on plastic surgery was comparably important. He modified the Langenbeck technique for cleft palate repair and developed, together with the gynaecologist G.A. Wagner, a technique of vaginoplasty.[1] Martin Kirschner published several articles on wound healing and infection and changed the current techniques of anaesthesiology when in 1931 he presented a technique of spinal anaesthesia, which was individually adjustable in dosage and level of anaesthesia. He personally performed 3500 operations with this technique in one year (1936). He also developed a technique of high-pressure local anaesthesia, which he used in 25 000 operations over 12 years. By means of compressed carbon dioxide or air the local anaesthetic was injected at a pressure or 2 bar using a specially designed apparatus.[1]

## MARTIN KIRSCHNER'S CONTRIBUTION TO ORTHOPAEDIC SURGERY

Wilhelm Conrad Roentgen's description of the principles of radiography in 1895 stimulated the development of orthopaedic surgery. The treatment of fractures up to then had not lacked invention or effort. Attempts to plate bones (Carl Hansmann, 1886) or join fractures by suturing predate the discovery of X-rays. But radiographic control often showed how badly fractures were aligned. This made surgeons think about how to improve treatment and results.

Using axially directed forces to reduce a fracture and retain an appropriate position was an idea dating back to the late 19^th^ century. For quite a long time plaster strips had been used to transmit the distracting force onto the bone often resulting in complications such as excruciating pain or skin necrosis. In 1908, however, the Swiss surgeon Fritz Steinmann (1872-1932) improved this technique by directing the realigning force directly onto the bone. His initial idea was to use a single perforating pin with a sharp tip which pierced the skin on both sides as it went in and out to transfix the bone in the transverse axis (what later became the “Steinmann-Pin”). Foreseeing the problem of infection when removing the pin he suggested in his original publication two pins inserted separately into the bone from both sides. Each pin only pierced the skin once. He did not mention his preliminary single-pin method. The same ingenious idea was developed by the Italian surgeon Codivilla in 1904; however this is not always recognized in German speaking countries.

The insertion of pins or wires into bone at this time was by using a hammer to exert a longitudinal force. The penetration of the pin into the cortical bone caused friction and inevitable rigid, unadjustable fixation of the metal ware. It was shown that the frictional forces could be overcome by fast rotation of the pin or wire and the introduction of the electric drill into orthopaedic surgery was very important. As long as the wire was rotating, its direction and position could be changed by the surgeon. When rotation stopped, the wire was rigidly fixed to the bone.

In this context it is important to mention the German surgeon Ernst Becker from Hildesheim. He proposed an electrical drill to insert a single 4 mm steel rod bicortically into the bone (in contrast to the initial bilateral approach of Steinmann). The use of the drill was thought to avoid further fracture dislocation and enhance precision of insertion.

The use of the single-pin technique gradually achieved acceptance in the early 20^th^ century and Fritz Steinmann was determined to claim the authorship of this idea. The 2^nd^ Balkan War in 1913 caused many shortages which led to a reduction in the material used in surgery. It was the German surgeon Rudolf Klapp who introduced the use of a thin, flexible wire for treatment of lower extremity fractures using traction. He burred a hole into the Calcaneum, through which the wire was inserted. In order to avoid direct surface-skin-bone contact the wire was directed towards the plantar surface and penetrated the skin there through separate incisions.

Up to this stage Kirschner had not contributed to the technique of applying traction directly to bone but when all these developments became available he realised the value of his method very quickly and published his first series of cases in 1909.

At this stage the surgical community was divided into two camps. On the one hand the use of the pin was favoured by some due to its better stability and rigid fixation within the bone. They also claimed that it was superior at sealing the pin-skin interface thus reducing infection. On the other hand wire-traction was thought to reduce damage to skin as well as bone. The wire could also be cut close to the shin on removal reducing the risk of infection. Martin Kirschner combined the advantages of both, wire and pin-extension techniques and made a significant contribution to the treatment of fractures.

**Figure 1:**
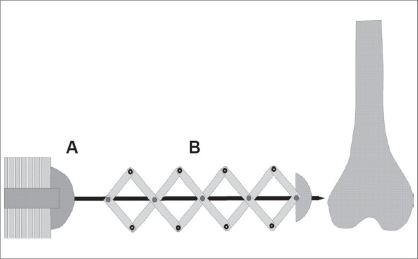
Schematic drawing of the apparatus used to drill flexible wires directly into the bone. The wire is mounted on the electrical drill (A) and passed through a folding grille instrument which prevents the wire from bending (redrawn according to Weiβer and Hörmann)

**Figure 2:**
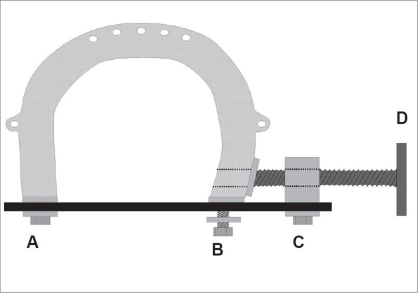
Schematic drawing of the device used to put the wire under tension. The wire is locked with screw A on the horseshoe and screw C on the lock. Turning the handle D puts the wire under tension. When adequate tension is accomplished, screw B is tightened. Finally, the lock C and the handle can be removed (redrawn according to Weiβer and Hörmann)

He inserted a thin wire directly into the bone, minimizing the size of the skin wounds and the damage to bone. He aimed to keep the wire fixed rigidly avoiding transverse wire movement. To achieve this, he used chromed, non-blazed piano-wire ranging from 0.7 to 1.5 mm in diameter. To prevent the wire bending he constructed an insertion device. He invented an instrument with a folding grille mechanism that stabilized the wire during insertion from the outside by holding it tight. The rigidity was maintained in the wire as the instrument was able to fold together as the wire advanced [Figure 1]. The problem of lateral movement and instability of a thin wire which causes pin track infection and osteomyelitis - could only be solved by wire tension. This problem had already been recognized and some unsatisfactory devices were available. Martin Kirschner, however, perfected the tension principle by constructing a horseshoe-shaped instrument [Figure 2]. The wire was rigidly fixed on one side and then tensioned with a T-shaped handle on the other side and fixed there with a screw like a coping saw.[3,4]

Inventing an adequate technique for the bone-wire interface was one problem Kirschner solved this way. Equally important, however, were the correct alignment and constant traction force on the bone in order to achieve the desired results. From his experience renovating or building two surgical clinics he was able to apply his knowledge to this challenge and in 1931 he presented his solutions. These were the extension bed to maintain constant traction over time and the extension cage. The latter was used to gain adequate fracture alignment by the use of traction forces. The important feature of the extension bed was a circular steel frame, onto which vertical steel rods could be attached. Those carried horizontally aligned rods with rollers. The position of these “gibbet-like” rods was adjustable and therefore the direction as well as the magnitude of the distracting force could be meticulously adjusted. But not only could axially directed traction forces be applied but also by using additional rollers transverse forces were possible and this improved the reduction of fractures. The head-end of the bed could be lowered to balance the traction forces thus avoiding moving the patient. The bed was designed like a trailer with a draw bar making transport easier over short distances.[5]

Even though the wire-distraction technique was commonly accepted in the 1940s it was Kirschner himself who abandoned parts of it. He reasoned that using a high- speed power drill may lead to heat induced damage resulting in infection or wire loosening. In order to avoid this he developed a wire-stapler, which was especially useful in times of war as it was easy to handle. This tube-like construction enclosed the entire wire, prevented it bending and enabled its insertion with a hammer.

The eponym Kirschner (K)-wire developed very quickly only a few years after the original publication. It can be found first in a paper by Müller in 1931 and came into common usage from 1932 onward.[3] Interestingly this eponym is much more commonly used in the English speaking world, whereas in German the wire are more often called “Bohrdrähte” (Bohr- means drill and -drähte translates for wires).

It is important to realize, however, that though Martin Kirschner developed the wire-technique almost to perfection he used it exclusively for traction treatment. The first paper suggesting the use of the wires for fracture fixation was published by Otto Loewe in 1932. Given the fact that this pre- dates Kirschner's death by 10 years, it is reasonable to assume that he was aware of this development but refused to use it in practice. Loewe realized the potential of the wires to act as spring-like stabilizing struts. He published one case using K-wires instead of the screw technique for femoral neck fractures. In the same year René Sommer (Dortmund, Germany) published a series of 20 cases. He described percutaneous wires to fix fractures with different patterns, (transverse, oblique and even complex), as well as dislocations of the acromio-clavicular joint. Loewe quotes advantages of this technique that are still valid at the present time: reducing implant bulk, avoiding excessive dissection, avoiding strangulation of bone (as in circlage wiring) and facilitating implant removal. Sommer also suggested the use of the wires for open reduction and internal fixation in difficult fractures. Another creative idea was the use of the wires as guidance for other implants. Sven Johansson presented a cannulated nail for treatment of femoral fractures in 1931. This was very similar to the Proximal Femoral Nail (Synthes) or to the Gamma-Nail (Stryker-Howmedica) used today, in which a temporary wire was used for precise positioning of the implant. Another application of the wire was introduced by Sterling Bunnell in the 1940s for joint transfixation in hand surgery. The final development of the K-wire was the technique of tension-band wiring. Today this is the standard technique for fractures of the olecranon and the patella as well as for internal fixation of finger joint arthrodesis. 2 K-wires inserted in a parallel manner and a figure 8 circlage wire transform traction forces into compression forces, a principle first described by Friedrich Pauwels.[3]

Very few techniques in surgery have had such a rapid, successful and long lasting impact as the use of wires in fracture treatment, either for traction or direct osteosynthesis. Due to its superior characteristics, the K-wire has remained basically unchanged in its design over the decades since it was introduced and it is a versatile tool in the hands of orthopaedic and plastic surgeons. Any arbitrary change of the eponym form Kirschner-to Loewe or Sommer-wire (L-wire, S-wire), despite the historical contributions of these authors, is definitely not recommended. Don't confuse your theatre nurse!
